# Niche and Range Shifts of *Aedes aegypti* and *Ae. albopictus* Suggest That the Latecomer Shows a Greater Invasiveness

**DOI:** 10.3390/insects14100810

**Published:** 2023-10-13

**Authors:** Peixiao Nie, Jianmeng Feng

**Affiliations:** College of Agriculture and Biological Science, Dali University, Dali 671003, China

**Keywords:** *Aedes aegypti*, *Aedes albopictus*, climate factor, invasiveness, niche shift, range shift

## Abstract

**Simple Summary:**

As the two major vectors of mosquito-borne pathogens, yellow fever (*Aedes aegypti*) and Asian tiger (*Ae. albopictus*) mosquitos are greatly threatening human health globally. However, their niche and range shifts remain little known. Using the largest occurrence record datasets to date, the present study examined the niche and range shifts between the native and invasive *Ae. aegypti* and *Ae*. *albopictus* populations. We detected substantial niche and range expansions in both species. Additionally, compared to the introduced *Ae. aegypti*, the more recent invader *Ae. albopictus* had greater niche and range expansions over its shorter invasion history, making it a more invasive vector of global mosquito-borne pathogens.

**Abstract:**

The yellow fever (*Aedes aegypti*) and Asian tiger (*Ae. albopictus*) mosquitos are major vectors of global mosquito-borne pathogens. However, their niche and range shifts, the underlying mechanisms, and related relative invasion rates remain scarcely known. We examined the niche and range shifts between the native and invasive *Ae. aegypti* and *Ae*. *albopictus* populations through dynamic niche and range models and the largest occurrence record datasets to date. We detected substantial niche and range expansions in both species, probably because the introduced populations have more opportunities to acclimate to diverse environmental conditions than their native counterparts. Mitigating climate change could effectively control their future invasions, given that future climate changes could promote their invasiveness. Additionally, compared to the introduced *Ae. aegypti*, the more recent invader *Ae. albopictus* had greater niche and range expansion over its shorter invasion history. In terms of the range shifts, *Ae. albopictus* had an invasion rate approximately 13.3 times faster than that of *Ae. aegypti*, making it a more invasive vector of global mosquito-borne pathogens. Therefore, considering its higher invasion rate, much more attention should be paid to *Ae. albopictus* in devising our strategies against prevailing global mosquito-borne pathogens than *Ae. aegypti*. Since small niche shifts could result in their large range shifts, niche shifts might be a more important indicator for biological invasion assessments.

## 1. Introduction

The yellow fever (*Aedes aegypti*) and Asian tiger (*Ae. albopictus*) mosquitos are two major vectors of mosquito-borne pathogens [1,2], including the viruses responsible for Zika virus disease, yellow fever, dengue fever, and chikungunya fever [3,4,5,6], which greatly threaten human health globally [7,8,9,10,11,12,13]. Native to Africa, *Ae. aegypti* has proliferated and spread into subtropical and tropical regions outside its native continent since the 16th and 17th centuries, a hectic time of global slave trade [14,15]. Unlike *Ae. aegypti*, *Ae. albopictus*, a mosquito native to Southeast Asia, has a shorter invasion history. It has proliferated and expanded its range from tropic and subtropical regions into temperate ones over the last 30–40 years [14,15,16]. However, studies comparing the invasion rates of the two *Aedes* species have been scarcely reported.

The ecological niche, which delimits environmental ranges under which a particular species could survive [17], is an essential conception in invasion ecology, probably due to its close associations with environmental conditions and the range shifts in alien invasive species under global change scenarios [18,19]. Recently, species distribution models (SDMs) have been widely used to project the potential ranges of alien invasive species and their shifts [20,21]. Nevertheless, one of their key presuppositions is the niche conservatism hypothesis, i.e., the alien invasive species conserves the niche inherited from its native counterpart [22,23]. However, until now, this hypothesis is still intensely debated. Some studies detected niche conservatism in alien invasive species [24,25,26,27], while others rejected it [28,29,30]. Niche shifts in alien invasive species might be an indicator of their invasiveness, i.e., alien invasive species with considerable niche expansions might have higher invasiveness. Thus, niche shifts in alien invasive species are also one of the important topics in invasion ecology [31,32,33]. However, to the best of our knowledge, few specific studies on the niche shift of *Ae. aegypti* and *Ae. albopictus* were reported.

Climatic factors, e.g., temperature and precipitation, are closely associated with the life histories of these mosquitos [34], including growth [35], development [36,37], reproduction [38,39], and even behavior [39,40]. For example, many studies have detected potential influences the temperature has on the life histories of these two *Aedes* species and their epidemiological features [41,42,43]. Notably, temperature could shorten the extrinsic incubation time (the time needed for a pathogen to develop inside *Ae. albopictus* or *Ae. aegypti* before it can be transmitted) [41,42]. These observations suggested that climatic factors could modify their roles as major vectors of global mosquito-borne pathogens [44]. Therefore, their invasion potentials and distribution patterns might be closely linked to their adaptability to changing climate conditions [45,46]. For example, Cunze et al. projected an increase in their suitable habitats in Europe under future climate change scenarios, in which their adaptability to climatic conditions played an essential role [47]. Recently, Laporta et al. predicted that future climate change could promote their proliferation at a global scale [46]. These findings undoubtedly offered important information for devising strategies against their future invasions. 

In addition to climate conditions, anthropogenic factors also play an important role in their potential ranges, probably because both have an inheritably anthropophilic nature [1,2,3,4]. Anthropogenic factors could affect their thermal regulation behaviors and exploitation ability for man-made habitats [48,49]. Therefore, studies on the influence of anthropogenic factors on their potential ranges have attracted much attention. For example, Dickens et al. observed that human and trade movement could facilitate range expansions in both vectors [50]. Abílio et al. found that the water container index significantly modified the ranges of both vectors in the urban/peri-urban regions of Mozambique [51]. Recently, Holeva-Eklund et al. observed that human population density was a principal predictor of the ranges of *Ae. aegypti* in Maricopa County, Arizona [52]. These studies have furthered our understanding of the roles anthropogenic factors have in determining the ranges of both vectors. 

Although climatic and anthropogenic factors could be responsible for their potential ranges, their relative roles in determining the ranges of these vectors remain debated. For example, while Dickens et al. found that anthropogenic factors had stronger associations with the potential ranges of both vectors than climatic factors [50], Liu et al. observed the opposite [45]. Therefore, the relative influences of anthropogenic and climatic factors on the potential ranges of *Ae. aegypti* and *Ae. albopictus* should be further investigated.

Topographical factors (e.g., elevation, slope, and aspect) are closely associated with the radiation, energy, and water conditions, forming various macro- and micro-habitats. Additionally, topographical patterns, such as deep canyons and lofty mountain ranges, could act as barriers against the spread of such invasive species [53,54,55]. Although the potential ranges of the two *Aedes* species might be strongly associated with climatic and anthropogenic factors, the effects of topographical patterns should not be neglected. However, the relative impacts of topographical, climatic, and anthropogenic factors on their potential ranges remain largely unknown.

The potential ranges of *Ae. aegypti* and *Ae. albopictus* have attracted considerable attention [46,56,57,58,59,60,61]. For instance, Kamal et al. predicted a marked increase in the potential ranges of *Ae. aegypti* across tropical and subtropical regions and an increase in the potential ranges of *Ae. albopictus* in the temperate regions of the United States and Europe based on future climate scenarios [62]. We hypothesized that since *Ae. aegypti* started its invasions about 300–400 years ago [14,15], it has a long time to adapt to novel conditions in the introduced regions. Therefore, there might be large range shifts between native and introduced *Ae. aegypti*, while smaller range shifts could be detected in *Ae. albopictus* due to its relatively shorter invasion history (30–40 years) [14,15,16]. However, most studies paid little attention to the range dynamics between the native and introduced *Aedes* species that might offer novel and essential information for controlling their invasions. For example, determining their potential range expansions (ranges only occupied by the introduced populations) during a certain period could provide essential information for assessing their invasiveness. Although *Ae. aegypti* and *Ae. albopictus* played major roles in the worldwide spread of chikungunya, dengue, and Zika fevers, their relative invasion rates in terms of the range shifts remain unknown.

In the present study, the niche and potential range shifts of an invasive species in its introduced regions relative to those of its native counterpart were used to calibrate its invasion potentials. Therefore, we grouped the population of each species into two populations: i.e., native and introduced populations, represented by those occurrences in native and introduced regions, respectively. We hypothesized that substantial niche and range shifts occurred between the native and introduced populations of each species. We developed models to detect niche and range shifts between native and introduced populations of each *Aedes* species and estimated the relative influences of climatic, anthropogenic, and topographical factors on their potential ranges and range shifts. Additionally, we also compared the invasion rates of the two *Aedes* species based on their range shifts. Our study could enrich and advance our understanding of the invasion potential of the two *Aedes* species.

## 2. Materials and Methods

### 2.1. Occurrence Record Datasets

**Figure 1:**
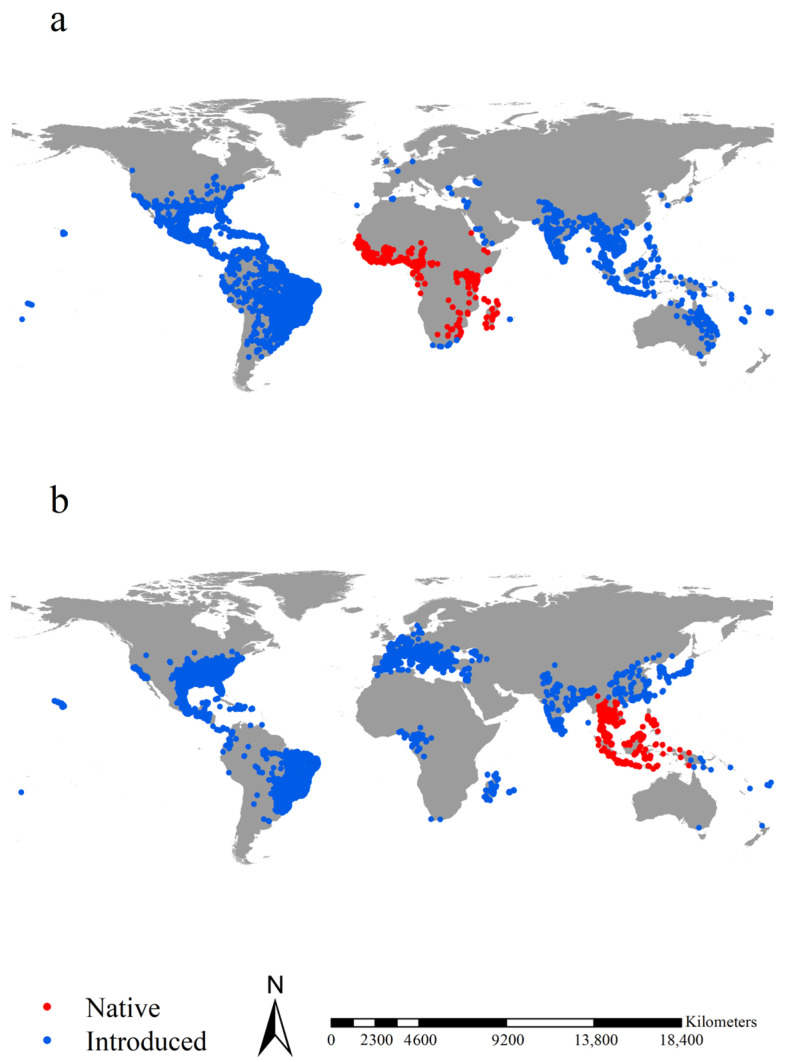
Occurrence records of the two *Aedes* species. Red and blue points in (**a**) indicated the native and introduced records of *Aedes aegypti*, respectively. Red and blue points in (**b**) indicated the native and introduced records of *Aedes albopictus*, respectively. After spatial rarefication, we retrieved 367 and 7239 native and introduced records for *Ae. aegypti*, respectively, and 460 and 7461 native and introduced records for *Ae. albopictus*, respectively, in total.

We retrieved the occurrence records of *Ae. aegypti* and *Ae. albopictus* from four sources: (1) the Global Biodiversity Information Facility (www.gbif.org, accessed on 2 March 2023), from which we obtained 60,197 and 78,619 records of *Ae. aegypti* and *Ae. albopictus*, respectively, with clear geographical coordinates; (2) a literature survey, primarily the paper by Kraemer et al. [63], from which we obtained 19,929 and 22,137 records with clear geographical coordinates for *Ae. aegypti* and *Ae. albopictus*, respectively; (3) the Early Detection and Distribution Mapping System (https://www.eddmaps.org/, accessed on 3 March 2023), from which we obtained 10,337 and 10,421 records of *Ae. aegypti* and *Ae. albopictus*, respectively; (4) the VectorMAP (https://vectormap.si.edu/, accessed on 4 March 2023), from which we obtained 37,545 and 42,167 occurrence records of *Ae. aegypti* and *Ae. albopictus*, respectively. Combined, our occurrence record datasets contained 128,008 and 153,344 records for *Ae. aegypti* and *Ae. albopictus*, respectively. We refine the occurrence record dataset by removing duplicate records and those with a geographical coordinate uncertainty of over 5 km, resulting in a dataset with 25,170 and 38,457 distinct records for *Ae. aegypti* and *Ae. albopictus*, respectively. We used SDMtoolbox by Brown et al. [64] to spatially rarefy the dataset with a radius of 5 km to reduce the effects of sampling bias on our models. Finally, we retained 7606 and 7921 occurrence records of *Ae. aegypti* and *Ae. albopictus*, respectively. Subsequently, we divided the dataset into four sub-datasets based on the native regions of *Ae. aegypti* and *Ae. albopictus* [65], i.e., 7239 records of *Ae. aegypti* in its introduced regions and 367 in its native regions and 7461 records of *Ae. albopictus* in its introduced regions and 460 in its native regions (Figure 1, Online datasets 1). The native and introduced occurrence records were input into species distribution models (SDMs) to project their native and introduced potential ranges, respectively.

### 2.2. Niche Dynamic Analysis

Following the COUE scheme (Centroid shift, Overlap, Unfilling, and Expansion) [66], we utilized ecospat, an R package for niche dynamic analysis [67], to calibrate the niche dynamics of the two *Aedes* species. We used occurrence records of the native and invasive *Ae. aegypti* and the spatial layers of the 32 predictors to extract values of each predictor for each occurrence record. Then, through an embedded principal component analysis (PCA) in the ecospat R package [66], we generated two PCA axes to delimit the niche space of *Ae. aegypti* in invaded and native regions. We divided the total environmental space into 100 × 100 grid cells [22,23,66]. We also utilized Kernel density functions to calibrate the smoothed density of occurrence records and available environment space along the first two PCA axes. According to the COUE scheme, the niche spaces were grouped into three elements: niche stabilized (S), niche unfilled (U), and niche expanded (E). Niche unfilled represented the niche space exploited only by the native *Ae. aegypti*; niche stabilized was the niche space exploited both by native and introduced *Ae. Aegypti*; and niche expanded indicated the niche space exploited only by introduced *Ae. aegypti*. The niche breadth of the native *Ae. aegypti* (*NB*) was the sum of S and U, and that of the introduced *Ae. aegypti* was the sum of S and E (*IB*). Breadth ratio (*BR*), indicating the ratio of the niche breadth of the introduced *Ae. aegypti* to that of the native *Ae. aegypti*, was calibrated as follows:

$$BR=\frac{IB}{NB}$$

If breadth ratio (*BR*) > 1, the niche breadth of the introduced *Ae. aegypti* was wider than that of the native counterpart and vice versa.

We also estimated the index of niche similarity (*SI*) to measure niche position shifts between the native and introduced *Ae. aegypti* as follows:

$$SI=\frac{2S}{IB+NB}$$

If index of niche similarity (*SI*) > 0.5, native and introduced *Ae. aegypti* occupied similar niche positions and vice versa. When index of niche similarity < 0.5 and breadth ratio > 1, the introduced *Ae. aegypti* did not conserve the niche inherited from its native counterpart, and the niche conservatism hypothesis was rejected [26]. In addition to the investigations on breadth ratio and index of niche similarity, we also conducted niche equivalency test and niche similarity test. 

Using this method, we also examined the niche dynamics between the native and introduced *Ae. aegypti* at the continental scale, separately, as well as those of *Ae. albopictus* at global and continental scales.

### 2.3. Predictors Used in SDMs

Our study retrieved 32 predictors to develop the SDMs, including 3 topographical, 10 anthropogenic, and 19 climatic predictors. As most (>85%) *Ae. aegypti* and *Ae. albopictus* occurrences were recorded after 1990, all predictors used in our study except the topographical ones had a time stamp of 1990–2020. We extracted a spatial elevation layer at a global scale from a digital elevation model (with a spatial resolution of 30 arc seconds) downloaded from Worldclim [68] and generated global spatial slope and aspect layers. The 1990–2020 anthropogenic predictors included eight land-use variables, gross domestic product per capita (GDP), and population density. The eight land-use predictors, retrieved from Land-Use Harmonization (http://luh.umd.edu/, accessed on 4 March 2023), had a spatial resolution of 0.25° and were divided into primary forested land, primary non-forested land, potentially secondary forested land, potentially secondary non-forested land, managed pasture, rangeland, urban land, and cropland. The GDP per capita and population density had a spatial resolution of 0.5 arc minutes and were retrieved from the Socioeconomic Data and Applications Center (https://sedac.ciesin.columbia.edu/, accessed on 4 March 2023). Instead of using the near-current (1970–2000) climatic predictors offered by Worldclim [69], we retrieved the most-updated monthly datasets of temperature and precipitation during 1990–2020 from the Climatic Research Unit (https://crudata.uea.ac.uk/, accessed on 6 March 2023) and applied the Biovars *R* package by Fick and Hijmans [69] to generate 19 climatic variables at a spatial resolution of 2.5 arc minutes. All 19 climatic variables were consistent with those in Worldclim [69]. Our 19-climatic-variable dataset included temperature and precipitation variables related to climatic factors at the month, quarter, and annual time scales. It must be noted that all 32 predictors were at or resampled into a spatial resolution of 2.5 arc minutes. 

We used the methodology proposed recently [70] to reduce the collinearity among the predictors. First, *biomod2* [71], an assembled SDM platform, was used to develop the preliminary SDMs and calibrate important values of each predictor (S1). We utilized VarImport, a function in *Biomod2*, to calculate predictors’ importance values calibrated by each algorithm in species distribution models [71]. Then, the averaged importance values of predictors were adopted. Subsequently, we used Pearson correlation analysis to calibrate the collinearity among the 32 predictors, using a threshold of >0.7 or <−0.7(S2) [72]. We retained the predictor with the higher importance values in each collinear pair. This process was repeated until no strong collinearity was observed. The retained predictors are presented in Table 1 and were included in the final SDMs to generate potential ranges for the two *Aedes* species and retrieve their importance values in the final SDMs.

### 2.4. Potential Ranges of the Two Aedes Species

We projected the potential ranges of the two *Aedes* species using *biomod2*, an assembled platform for SDMs [71]. We used the retained predictors and adopted nine algorithms from *biomod2* to project the potential ranges of *Ae. aegypti* and *Ae. albopictus*. The nine algorithms included Artificial Neural Network, Classification Tree Analysis, Generalized Linear Model, Flexible Discriminant Analysis, Multiple Adaptive Regression Splines, Generalized Boosting Model, Random Forest for Classification and Regression, Maximum Entropy Modeling, and Surface Range Envelope. Only algorithms showing an area under the curve (AUC) > 0.8 or true skill statistic (TSS) > 0.6 were included in the assembled SDMs to obtain reliable and central tendencies of the potential ranges’ projections [73]. A weight proportional to their TSS evaluation was given to each model’s projection [71]. As required by presence-only SDMs, we conducted a three-time random selection at a global terrestrial scale (except Antarctica) as follows: equal numbers of pseudo absences when the number of real occurrence records was over 1000, or 1000 pseudo absences randomly were selected, following Cao et al. [74]. We used the maximization sensitivity–specificity sum thresholds to calibrate the potential ranges of the two *Aedes* species, as suggested by Liu et al. (2016) [75].

To evaluate the reliability of SDMs, a five-time cross-validation was utilized, in which we randomly selected 70% of the total occurrences to develop the SDMs and used the remaining 30% to calibrate the models’ reliability [71]. We also applied null models to evaluate our model’s reliability following a robust methodology [76,77], in which we randomly selected virtual occurrence records (equal in number to 70% of the total occurrences) to generate SDMs and used 30% of the real occurrences to assess the null SDMs’ performances (S3). 

### 2.5. Range Shifts of the Two Aedes Species

We decomposed the total potential ranges of *Ae. aegypti* into three parts, following Yang et al. [78]: range expansion (*RE*), potential ranges only occupied by introduced *Ae. aegypti*; range stability (*RS*), potential ranges shared by native and introduced *Ae. aegypti*; and range unfilling (*RU*), potential ranges occupied by native *Ae. aegypti* only. The potential ranges of native *Ae. aegypti* (*PRN*) were the sum of *RS* and *RU*, whereas those of introduced *Ae. aegypti* (*PRI*) were the sum of *RE* and *RS*. We constructed a range ratio index (*RRI*) to compare the *Ae. aegypti PRI* and *PRN* as follows:

$$RRI=\frac{PRI}{PRN}$$

When the potential ranges of the introduced *Ae. Aegypti* (*PRI*) were greater than its native counterpart (*PRN*), the range ratio index (*RRI*) was >1 [78,79].

We also constructed a range similarity index (*RSI*) to calibrate the range position shifts between the introduced and native *Ae. aegypti*, rendered as follows:

$$RSI=\frac{2RS}{PRI+PRN}$$

When the native and introduced *Ae. aegypti* were in similar range positions, the range similarity index (*RSI*) was >0.5 [78,79].

Similar methods were adopted to calibrate the range shifts between the introduced and native *Ae. albopictus*.

## 3. Results

### 3.1. Major Predictors for the Potential Ranges

Our results showed that SDMs calibrated by Artificial Neural Network, Classification Tree Analysis, Generalized Linear Model, Flexible Discriminant Analysis, Multiple Adaptive Regression Splines, Generalized Boosting Model, Random Forest for Classification and Regression and Maximum Entropy Modeling were included in the assembled SDMs, whereas those calibrated by Surface Range Envelope were removed because their AUC and TSS were less than 0.8 and 0.6, respectively (S4). The importance values of the most important predictors of potential ranges in decreasing order were isothermality (0.351), GDP per capita (0.196), and population density (0.113) for native *Ae. aegypti*, temperature seasonality (0.369), GDP per capita (0.194), and population density (0.112) for introduced *Ae. aegypti*, mean annual temperature (0.230), Temperature seasonality (0.197), and annual precipitation (0.195) for native *Ae. albopictus*, and the mean temperature of the warmest quarter (0.150), temperature seasonality (0.149), and precipitation of the wettest month (0.108) for introduced *Ae. albopictus* (Table 1 and Table S5). Our results also indicated that none of the topographical predictors had an importance value in the top ten list for any of the potential ranges (Table 1). While GDP per capita and population density were the second and third most important predictors in the SDMs for *Ae. aegypti*, they were not in the top three among the SDMs for *Ae. albopictus*. In summary, climatic predictors showed the highest importance values for all potential ranges, followed by anthropogenic and topographical factors (Table 1).

### 3.2. Niche Dynamics of the Two Aedes Species

The global scale investigations into niche dynamics of *Ae. aegypti* showed that at the global scale, the niche expanded, niche stabilized, and niche unfilled were 0.0045, 0.955, and 0.027, respectively; the breadth ratio and niche similarity index were 1.018 and 0.964, respectively (Table 2). Therefore, at the global scale, the niche shifts in *Ae. aegypti* supported the niche conservatism hypothesis. At the continental scale, smaller niche shifts were detected between the native *Ae. aegypti* and the introduced counterpart in South America relative to those between the native *Ae. aegypti* and the introduced counterparts in Asia and North America (Table 2). At the global scale, the niche expanded, niche stabilized, and niche unfilled of *Ae. albopictus* were 0.382, 0.618, and 0.101, respectively; the breadth ratio and niche similarity index were 1.391 and 0.719, respectively (Table 2). Therefore, at the global scale, the introduced *Ae. albopictus* conserved the niche inherited from its native counterpart. At the continental scale, relatively larger niche expanded, niche stabilized, and niche unfilled were detected between the native *Ae. albopictus* and the introduced counterparts in Europe, South America, and Europe, respectively (Table 2). Additionally, all of our equivalency tests and similarity tests showed that the two introduced *Aedes* species have niches equivalent to their native counterparts but similar by chance, which, to a certain extent, was consistent with our observations on their shifts in breadth ratio and niche similarity, which indicated the niche conservatism of the two introduced *Aedes* species.

### 3.3. Potential Ranges of the Two Aedes Species

All four SDMs for the potential ranges showed high performance. Our SDMs for projecting the potential ranges of native and introduced *Ae. aegypti* had AUCs of 0.993 and 0.984 and TSS values of 0.936 and 0.878 (S3). The SDMs for projecting the potential ranges of native and introduced *Ae. albopictus* had AUCs of 0.997 and 0.978 and TSS values of 0.967 and 0.852 (S3). All four SDMs performed better than the null SDMs (all *p* < 0.001) (S3). The maximization sensitivity–specificity sum thresholds for calibrating the potential ranges were 0.58 and 0.45 for introduced and native *Ae. aegypti* and 0.50 and 0.47 for introduced and native *Ae. albopictus*, respectively. 

**Figure 2:**
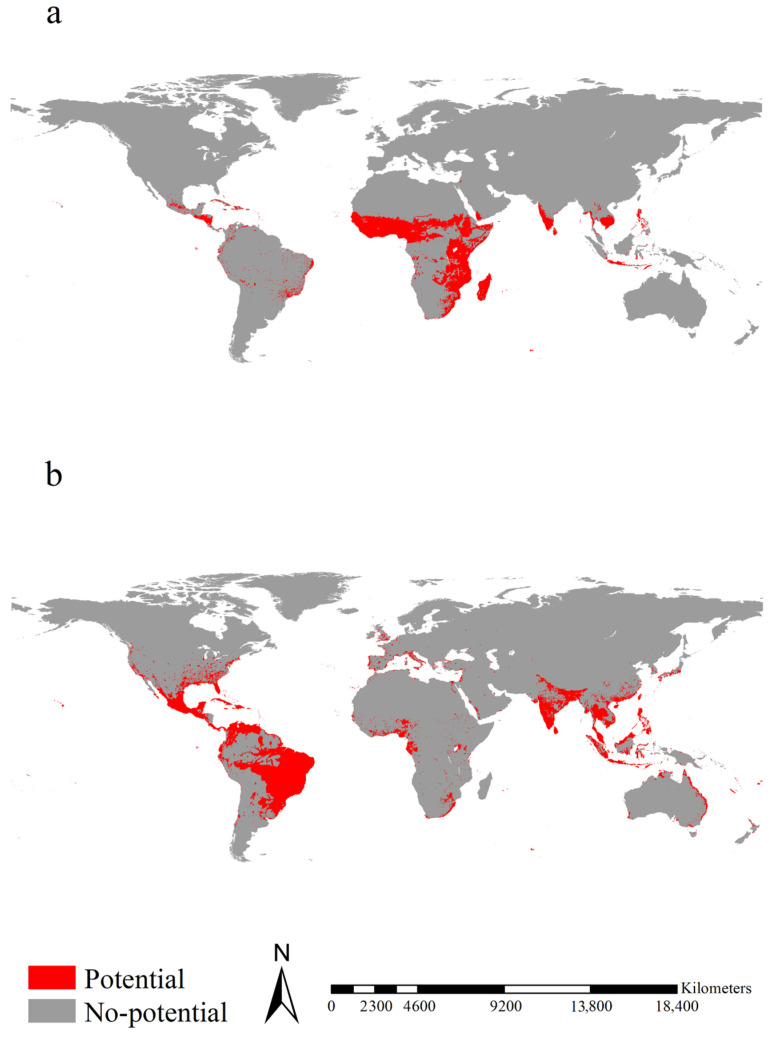
Potential ranges of *Aedes aegypti*. (**a**,**b**) represented the potential ranges of the native and introduced *Ae. aegypti*, respectively. The potential ranges of native *Ae. aegypti* were mainly observed in Cambodia, Cameroon, Central Africa, Chad, East Africa from Ethiopia and Somalia to South Africa, Honduras, Indonesia, Madagascar, Mexico, Nicaragua, South India, Sri Lanka, the Philippines, tropical West Africa, and Vietnam. The potential ranges of introduced *Ae. aegypti* were mainly detected in Bangladesh, Brazil, Colombia, India, Indonesia, Japan, Malaysia, Nepal, Thailand, the Philippines, the southeastern coastal regions of China, and vast regions of Mexico and Venezuela. The potential ranges were calibrated by eight algorithms, i.e., Artificial Neural Network, Classification Tree Analysis, Generalized Linear Model, Flexible Discriminant Analysis, Multiple Adaptive Regression Splines, Generalized Boosting Model, Random Forest for Classification and Regression, and Maximum Entropy Modeling, and a weight proportional to their TSS evaluation was given to each model’s projection.

**Figure 3:**
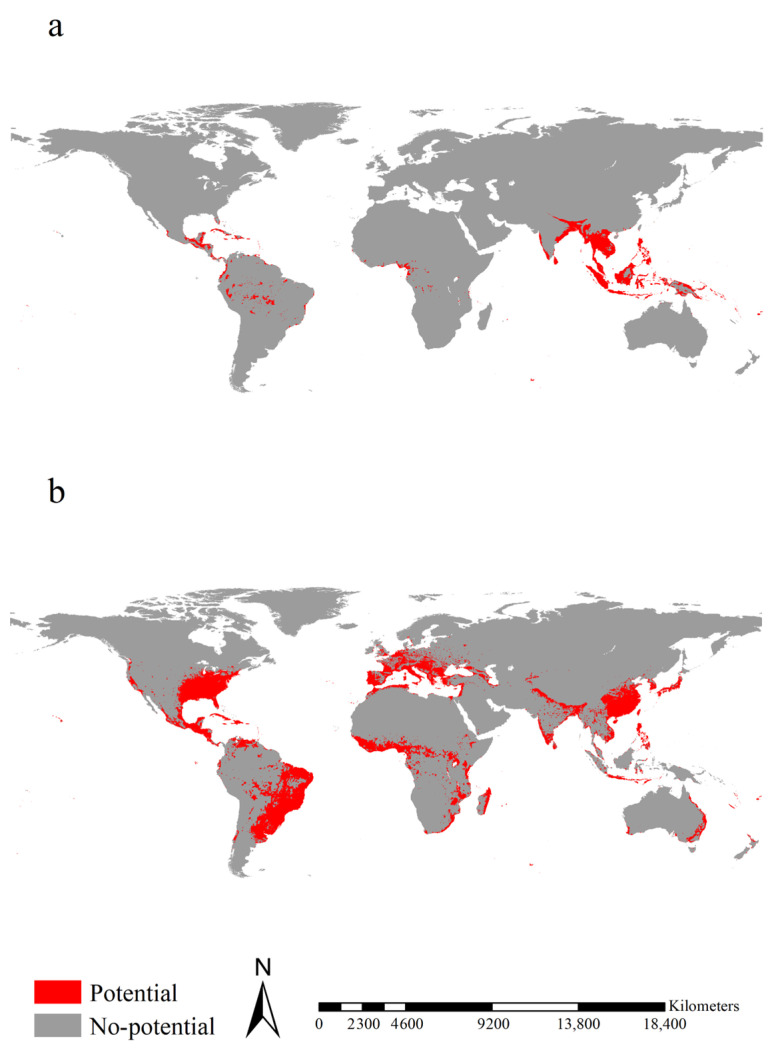
Potential ranges of *Aedes albopictus*. (**a**,**b**) represented the potential ranges of the native and introduced *Aedes* albopictus, respectively. The potential ranges of native Ae. Albopictus were mainly observed in Bangladesh, Bolivia, Brazil, Cambodia, Ecuador, India, Indonesia, Laos, Malaysia, Mexico, Sri Lanka, Thailand, and Vietnam. The potential ranges of introduced *Ae. Albopictus* were mainly observed in Cambodia, eastern China, eastern United States, Europe, India, Indonesia, Japan, Mexico, the Philippines, tropical regions of West Africa, and Vietnam. The potential ranges were calibrated by eight algorithms, i.e., Artificial Neural Network, Classification Tree Analysis, Generalized Linear Model, Flexible Discriminant Analysis, Multiple Adaptive Regression Splines, Generalized Boosting Model, Random Forest for Classification and Regression, and Maximum Entropy Modeling, and a weight proportional to their TSS evaluation was given to each model’s projection.

Potential ranges for native *Ae. aegypti* were mainly observed in Cambodia, Cameroon, Central Africa, Chad, East Africa from Ethiopia and Somalia to South Africa, Honduras, Indonesia, Madagascar, Mexico, Nicaragua, South India, Sri Lanka, the Philippines, tropical West Africa, and Vietnam, covering 9.38 × 10^6^ km^2^ (Figure 2a). Potential ranges of introduced *Ae. aegypti* were detected in Bangladesh, Brazil, Colombia, India, Indonesia, Japan, Malaysia, Nepal, Thailand, the Philippines, the southeastern coastal regions of China, vast regions of Mexico and Venezuela, covering 14.53 × 10^6^ km^2^ (Figure 2b). Potential ranges for native *Ae. albopictus* were mainly projected in Bangladesh, Bolivia, Brazil, Cambodia, Ecuador, India, Indonesia, Laos, Malaysia, Mexico, Sri Lanka, Thailand, and Vietnam, covering 4.71 × 10^6^ km^2^ (Figure 3a). Potential ranges of introduced *Ae. albopictus* were mainly projected in Cambodia, eastern China, eastern United States, Europe, India, Indonesia, Japan, Mexico, the Philippines, tropical regions of West Africa, and Vietnam, covering 18.01 × 10^6^ km^2^ (Figure 3b). 

### 3.4. Range Shifts of the Two Aedes Species

**Figure 4:**
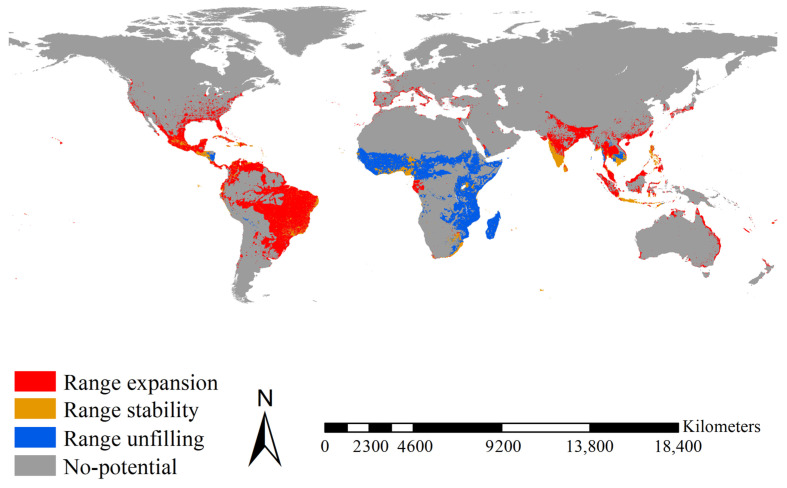
Range shifts between the native and introduced *Aedes aegypti*. Range expansions between native and introduced *Ae. aegypti* were mainly detected in Bangladesh, Brazil, Colombia, Guatemala, India, Indonesia, Malaysia, Mexico, southeastern United States, Thailand, and Venezuela. Range stability between native and introduced *Ae. aegypti* was mainly detected in Honduras, Cameroon, Togo and Nigeria, South Africa, India, Sri Lanka, and Vietnam. Range unfilling was mainly anticipated in Madagascar, the tropical regions of East Africa from Ethiopia and Somalia to South Africa and West Africa. Red, blue, and orange indicated the range expanded, range unfilled, and range stabilized, respectively.

Range expansion for *Ae. aegypti* was mainly projected for Bangladesh, Brazil, Colombia, Guatemala, India, Indonesia, Malaysia, Mexico, the southeastern United States, Thailand, and Venezuela, covering 12.03 × 10^6^ km^2^ (Figure 4). Range stability for *Ae. aegypti* was mainly projected for Cameroon, Honduras, India, Nigeria, South Africa, Sri Lanka, Togo, and Vietnam, covering 2.50 × 10^6^ km^2^ (Figure 4). Range unfilling was mainly anticipated in the tropical regions of East Africa from Ethiopia and Somalia to South Africa, Madagascar, and West Africa, covering 6.88 × 10^6^ km^2^ (Figure 4). Accordingly, the range ratio index was 1.549, i.e., the introduced *Ae. aegypti* had 1.55 times more potential ranges than the native one. The range similarity index was 0.209, i.e., the native and introduced *Ae. aegypti* occupied different range positions.

**Figure 5:**
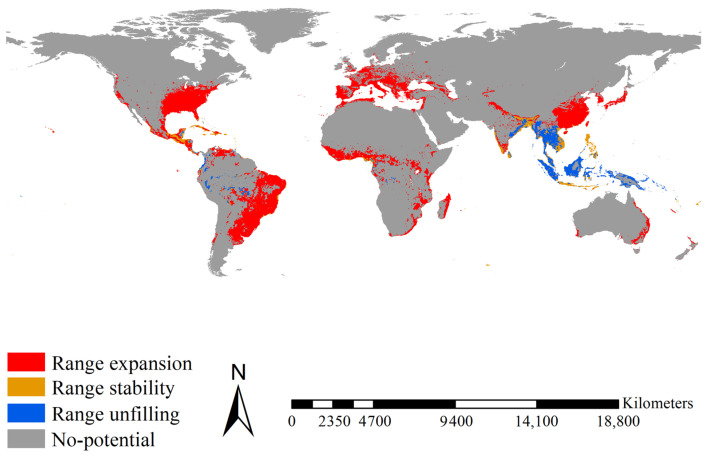
Range shifts between the native and introduced *Ae. Albopictus*. Range expansions between native and introduced *Ae. Albopictus* were mainly detected in Brazil, eastern China, Europe, India, Japan, Madagascar, Mexico, the eastern United States, and the tropical regions of East Africa. Range stability between native and introduced *Ae. Albopictus* was mainly detected in Bangladesh, India, Indonesia, Mexico, and the Philippines. Range unfilling was mainly projected in India, Indonesia, Malaysia, and Thailand. Red, blue, and orange indicated the range expanded, range unfilled, and range stabilized, respectively.

Range expansion for *Ae. albopictus* was mainly projected for Brazil, eastern China, Europe, India, Japan, Madagascar, Mexico, the eastern United States, and the tropical regions of East Africa, covering 16.10 × 10^6^ km^2^ (Figure 5). Range stability for *Ae. albopictus* was mainly projected for Bangladesh, India, Indonesia, Mexico, and the Philippines, covering 1.91 × 10^6^ km^2^ (Figure 5). Range unfilling was mainly anticipated for India, Indonesia, Malaysia, and Thailand, covering 2.80 × 10^6^ km^2^ (Figure 5). Accordingly, the range ratio index was 3.824, i.e., the introduced *Ae. albopictus* had ca. 3.83 times more potential ranges than the native one. The range similarity index was 0.168, i.e., the native and introduced *Ae. albopictus* occupied different range positions. Additionally, as a more recent invader, introduced *Ae. albopictus* showed a larger range expansion (16.10 × 10^6^ km^2^ vs. 12.03 × 10^6^ km^2^, ca. 1.33 times) than the introduced *Ae. aegypti* over its shorter invasion history (ca. one tenth). Therefore, in terms of range shifts, *Ae. albopictus* had an invasion rate ca. 13.3 times that of *Ae. aegypti*.

## 4. Discussion

Our study detected substantial niche and range shifts that occurred between the native and introduced populations of both species. We observed stronger roles of climatic factors in their potential ranges relative to those of anthropogenic and topographical ones. Our study also suggested that in terms of the niche and range shifts, *Ae. albopictus*, as a latecomer, exhibited higher invasiveness than *Ae. aegypti*. Therefore, our study could enrich and further our understanding of their invasion potential and risk assessment.

Although the niche conservatism hypothesis on alien invasive species has received much attention in the past decades, this hypothesis is still under intense debate. Recently, Liu et al. (2020) argued that most invasive species largely conserve their climatic niche [26], which, to a great extent, supported our observations that the two introduced *Aedes* species conserved the niche spaces inherited from their native counterparts. *Ae. albopictus* had relatively shorter invasions than *Ae. aegypti*, i.e., 30–40 vs. 300–400 years [14,15,16]. However, our study showed that compared with *Ae. aegypti, Ae. albopictus* had larger niche expansions (0.382 vs. 0.045), indicating that the latter has evolved stronger adaptability to novel environmental conditions in a relatively shorter history and has higher invasiveness. Our study also showed that the niche dynamics of the two *Aedes* species differed among continents. For example, larger niche expansions of *Ae. albopictus* were detected in Europe, while smaller ones were observed in South America (0.499 vs. 0.022). The introduced *Ae. albopictus* in Europe and South America survived in temperate and tropical regions, respectively, and the native *Ae. albopictus* originated from tropical regions in Asia. Therefore, climatic differences between the native *Ae. albopictus* and the introduced counterpart in Europe were larger than those between the native *Ae. albopictus* and the introduced counterpart in South America, resulting in larger niche expansions. Additionally, previous studies have shown that *Ae. albopictus* had strong adaptability to novel environmental conditions. For example, Lacour et al. (2015) detected adaptive synchronization of the diapause process of *Ae. albopictus* in adverse winter conditions [80]. Marini et al. (2020) observed the ability of *Ae. albopictus* to quickly adapt to colder environments [81]. Therefore, the differences in niche dynamics among the continents might not only be closely associated with the climatic differences between the regions where the introduced and native *Ae. albopictus* survived, but also with a strong adaptability to novel conditions.

Over the last decades, various studies have examined the potential ranges or range shifts of *Ae. aegypti* and *Ae. albopictus* [45,47,62,82,83,84,85]. However, even though most of the occurrences were recorded after 1990, most previous studies used the near-current condition climatic datasets (1970–2000) from Worldclim [69] to probe the range shifts of the two *Aedes* species [46,56]. Conversely, we constructed the climatic factors’ datasets for 1990–2020, potentially achieving a better match between the climatic datasets and the occurrence records. Additionally, most previous global-scale studies retrieved far fewer occurrence records than we did, even though they did their best to retrieve as many occurrence records as possible. First, most of them built datasets without sampling bias correction with fewer records than our study, e.g., 19,930 records of *Ae. aegypti* and 22,137 *Ae. albopictus* [5], 9735 *Ae. aegypti* and 13,093 *Ae. albopictus* [46], 19,930 *Ae. aegypti* and 22,137 *Ae. albopictus* [50], 4251 *Ae. aegypti* and 3341 *Ae. albopictus* [62], and 6599 *Ae. albopictus* [82]. However, the present study built a dataset without sampling bias correction with 25,170 and 38,457 distinct occurrence records for *Ae. aegypti* and *Ae. albopictus*, respectively. To our knowledge, this was the largest occurrence record dataset of the two *Aedes* species. Second, most of them built sampling bias-corrected datasets with fewer records than our study, e.g., 2303 *Ae. aegypti* and 1427 *Ae. albopictus* [62] and 673 *Ae. albopictus* [82]. Additionally, we developed a sampling bias-corrected dataset with 7606 and 7921 occurrence records of *Ae. aegypti* and *Ae. albopictus*, respectively. Moreover, we constructed SDMs using nine algorithms in *Biomod2* [71] to probe their range shifts with the advantage of reducing the arbitrariness of fewer algorithms. Therefore, the present study might be more reliable than previous studies due to its large dataset, time-matching between climatic predictors and occurrence records, and the use of diverse algorithms. However, we have to acknowledge that the potential ranges in our study are just the results of theoretical projections without biotic factors in the SDMs. Therefore, our projections might not be fully consistent with the realized ranges of the two *Aedes* species, and further investigations should be needed in the future.

In 2018, Dickens et al. predicted that the potential ranges of the two *Aedes* species were similar and restricted mainly to the subtropical and tropical regions, with the range of *Ae. albopictus* extending further into higher latitudes [50]. Recently, Laporta et al. projected that the ranges of the two *Aedes* species would expand in the Northern Hemisphere and shrink in the Southern Hemisphere [46]. Although these relevant studies enhanced our understanding of their potential invasiveness, the present study advances this knowledge even further. Unlike these previous studies, we built models to examine range shifts between the native and introduced *Ae. aegypti* and *Ae. albopictus*. We identified their respective potential ranges (*PRIs* and *PRNs*), estimated their ratios (*RRIs*), and examined the effects of controlling factors. Moreover, our study examined the range expansions of the two introduced *Aedes* species and compared their invasion rates. Therefore, our study offers some novel and important information on the invasiveness of the investigated *Aedes* species. 

Our study showed that climatic factors had a stronger influence on the potential ranges of the two *Aedes* species than anthropogenic and topographical factors. This finding suggested that although anthropogenic factors such as population density and GDP per capita could modify their exploitation of man-made habitats [48,49], their ability to occupy potential ranges might be determined by their adaptation to the local climatic conditions. Additionally, well-developed transportation networks might shadow the barrier effects of topographical factors such as huge and lofty mountain ranges and deep valleys. These observations suggested that climate change mitigation could effectively control the potential invasions of the two *Aedes* species [45,62]. Although we observed important roles for climatic factors in predicting the potential ranges of the two *Aedes* species, the GDP per capita and population density played a stronger role in determining the potential ranges of *Ae. aegypti* than *Ae. albopictus*. This difference might be because while *Ae. aegypti* feeds primarily on humans [86], *Ae. albopictus* feeds on diverse mammalian and avian species [48,87,88,89].

Sirami et al. (2017) argued that the relative effects of anthropogenic and climatic factors on the potential ranges of invasive species largely depend on the spatial scale used [90], i.e., a stronger role is found for climatic factors at large scales and a stronger role for anthropogenic factors at small scales, somewhat supporting the findings of Liu et al. [45] and Ding et al. [91] and our observation of a stronger role for climatic factors at the global scale. However, Dickens et al., who also investigated at a global scale, found that anthropogenic factors had a stronger role than climatic factors in determining the potential ranges of both vectors [50]. Additionally, a small-scale field study by Tsuda et al. [92] detected a stronger role for climatic predictors than anthropogenic ones in determining the ranges of the two vectors in three villages in northern Thailand. Conversely, a small-scale field study by Holeva-Eklund et al. found that population density, an anthropogenic factor, had a stronger effect on the potential ranges of *Ae. aegypti* than climatic ones [52]. Therefore, the argument that the relative roles of anthropogenic and climatic factors in determining the potential ranges depended on the spatial scale might not be a general pattern, leaving their relative effects on determining the potential ranges of the two *Aedes* species under debate and in need of further investigation.

Although both *Aedes* species are competent vectors of several diseases, their invasion history durations differ. The global invasions of *Ae. aegypti* started about 300–400 years ago, triggered by the global slave trade in the 16th and 17th centuries [14,15], whereas it started only 30–40 years ago for *Ae. albopictus* induced by accidental introduction [14,16]. Our study showed that the range expansion of *Ae. albopictus* was larger than that of *Ae. aegypti* (16.10 vs. 12.03 × 10^6^ km^2^, i.e., 1.33 times larger) in a relatively shorter invasion history (ca. one tenth). Therefore, from a range shift perspective, the invasion rate of *Ae. albopictus* was about 13.3 times higher than that of *Ae. aegypti*. This finding was somewhat supported by a consensus report arguing that *Ae. albopictus* was the most invasive mosquito in the world [93] and one of the world’s 100 worst invasive species [94], whereas *Ae. aegypti* was not. The higher invasion rate of *Ae. albopictus* might be due to the rapid growth of international trade in used tires over the past decades [95] and its strong ecophysiological plasticity [96]. However, we must acknowledge that invasion rates of *Ae. albopictus* and *Ae. aegypti* may be high at first and then drop and remain more stable at the final stage. *Ae. albopictus* might be in the first stage with a high invasion rate, whereas *Ae. aegypti* might be at the final stage, showing a stable and low invasion rate. Therefore, our observation of the invasion rates concerned just overall invasion rates across all stages up to now and could not reflect temporal variations of invasion rates across all stages. 

Potential ranges of *Ae. aegypti* and *Ae. albopictus* and their shifts have attracted much attention in the past decades [52,62]. Undoubtedly, these studies have offered important information for devising strategies to fight their invasions. For example, Laporta et al. found that under future climate change scenarios, the potential ranges of *Ae. aegypti* and *Ae. albopictus* might expand in the Northern Hemisphere, whereas in the Southern Hemisphere, their potential range might show a decreasing trend, suggesting stricter strategies against their invasions should be needed in the Northern Hemisphere [46]. At the global scale, we detected a larger range ratio in *Ae. aegypti* and *Ae. albopictus* relative to their niche breadth ratios, i.e., 1.549 vs. 1.018 and 3.824 vs. 1.391, respectively. This might indicate that small niche shifts in them could induce their large range shifts and that niche shifts might be a more important indicator for biological invasion assessments [74].

## 5. Conclusions

Our study detected substantial niche and range expansions in introduced *Ae. aegypti* and *Ae. albopictus* relative to their respective native counterparts, probably because the introduced populations have much more opportunities to adapt to novel climatic conditions. Climate change mitigation could effectively control their invasions, given that climatic factors played strong roles in determining their potential ranges, and future climate changes could promote their invasions. *Ae. albopictus* underwent larger niche range expansions over its relatively short invasion history than *Ae. aegypti*. In terms of the niche and range shifts, *Ae. albopictus* had an invasion rate about 13.3 times faster than that of *Ae. aegypti*. Therefore, compared with *Ae. aegypti*, the niche and range shifts of *Ae. albopictus* suggested that the latecomer showed higher invasiveness over its relatively shorter invasion history. Since small niche shifts in them could induce their large range shifts, niche shifts might be a more important indicator for biological invasion assessments.

## Figures and Tables

**Table 1:** The importance values of the retained predictors in the final species distribution models for *Ae. aegypti* and *Ae. albopictus*.

*Aedes aegypti*						*Aedes albopictus*					
Native *Ae. aegypti*			Introduced *Ae. aegypti*			Native *Ae. albopictus*			Introduced *Ae. albopictus*		
Category	Predictors	Importance Values	Category	Predictors	Importance Values	Category	Predictors	Importance Values	Category	Predictors	Importance Values
Climate	Bio3	0.351	Climate	Bio4	0.369	Climate	Bio1	0.230	Climate	Bio10	0.150
GDP	GDP	0.196	GDP	GDP	0.194	Climate	Bio4	0.197	Climate	Bio4	0.149
POP	POP	0.113	POP	POP	0.112	Climate	Bio12	0.195	Climate	Bio13	0.108
Climate	Bio7	0.052	Climate	Bio10	0.099	Climate	Bio18	0.148	Land-use	Pastr	0.082
Land-use	Primf	0.030	Climate	Bio9	0.096	POP	POP	0.136	Land-use	Urban	0.056
Land-use	Range	0.028	Land-use	Crop	0.045	Land-use	Range	0.059	POP	POP	0.056
Climate	Bio13	0.024	Climate	Bio13	0.033	Climate	Bio5	0.050	Climate	Bio17	0.050
Land-use	Secdn	0.024	Land-use	Primf	0.015	GDP	GDP	0.049	Climate	Bio18	0.03
Land-use	Primn	0.020	Land-use	Urban	0.011	Land-use	Pastr	0.032	Land-use	Crop	0.032
Climate	Bio1	0.019	Climate	Bio19	0.011	Land-use	Crop	0.021	Climate	Bio8	0.030
Climate	Bio14	0.017	Topography	Ele	0.010	Land-use	Primf	0.018	Climate	Bio19	0.028
Land-use	Pastr	0.015	Climate	Bio18	0.010	Topography	Ele	0.018	Land-use	Primf	0.025
Climate	Bio18	0.012	Climate	Bio17	0.008	Climate	Bio14	0.018	Topography	Ele	0.022
Land-use	Crop	0.011	Land-use	Pastr	0.007	Topography	Slop	0.011	Land-use	Range	0.020
Climate	Bio15	0.007	Land-use	Range	0.007	Climate	Bio2	0.007	Land-use	Primn	0.016
Topography	Slop	0.007	Land-use	Primn	0.007	Land-use	Secdn	0.003	Climate	Bio2	0.010
Land-use	Secdf	0.004	Topography	Slop	0.005	Land-use	Secdf	0.003	Land-use	Secdn	0.010
Topography	Asp	0.003	Land-use	Secdf	0.004	Land-use	Urban	0.003	Topography	Slop	0.007
Land-use	Urban	0.002	Land-use	Secdn	0.003	Land-use	Primn	0.002	Land-use	Secdf	0.005
			Topography	Asp	0.003	Topography	Asp	0.002	Topography	Asp	0.001
			Climate	Bio2	0.002						

Note: Bio1: annual mean temperature (°C); Bio2: mean diurnal range (°C); Bio3: isothermality; Bio4: temperature seasonality; Bio5: max temperature of the warmest month (°C); Bio7: temperature annual range (°C); Bio8: mean temperature of the wettest quarter (°C); Bio9: mean temperature of the driest quarter (°C); Bio10: mean temperature of the warmest quarter (°C); Bio12: annual precipitation (mm); Bio13: precipitation of the wettest month (mm); Bio14: precipitation of the driest month (mm); Bio15: precipitation seasonality (mm); Bio17: precipitation of the driest quarter (mm); Bio18: precipitation of the warmest quarter (mm); Bio19: precipitation of the coldest quarter (mm); Asp: aspect (°); Ele: elevation (m); Slop: slope (°); Urban: fractions of urban land; Primf: fractions of forested primary land; Crop: fractions of cropland; Pastr: fractions of managed pasture; Secdf: fractions of potentially forested secondary land; Range: fractions of rangeland; Primn: fractions of non-forested primary land; Secdn: fractions of potentially non-forested secondary land; GDP: gross domestic product; and POP: population density. The importance values were calibrated by eight algorithms, i.e., Artificial Neural Network, Classification Tree Analysis, Generalized Linear Model, Flexible Discriminant Analysis, Multiple Adaptive Regression Splines, Generalized Boosting Model, Random Forest for Classification and Regression, and Maximum Entropy Modeling, and the averaged importance values of each predictor were adopted.

**Table 2:** Niche dynamics of the two *Aedes* species.

Species (Native Range)	Introduced Population	Expan	Stable	Unfill	Breadth	EquaT	Similar	SimiT
*Aedes aegypti* (Africa)	Global	0.045	0.955	0.027	1.017	ns	0.964	ns
	Asia	0.087	0.913	0.005	1.090	ns	0.952	ns
	North America	0.137	0.863	0.058	1.085	ns	0.899	ns
	South America	0.008	0.992	0.148	0.877	ns	0.927	ns
*Ae. albopictus* (Asia)	Global	0.382	0.618	0.101	1.391	ns	0.719	ns
	Africa	0.055	0.945	0.208	0.867	ns	0.878	ns
	Europe	0.499	0.501	0.806	0.765	ns	0.434	ns
	North America	0.332	0.668	0.359	0.974	ns	0.659	ns
	South America	0.022	0.978	0.286	0.791	ns	0.864	ns

Note: EquaT: equivalency test; SimiT: similarity test; Epan: niche expanded; Stable: niche stabilized; Unfill: niche unfilled; Breadth: breadth ratio; and Similar: similarity index. We utilized the COUE scheme to calibrate niche dynamics of the two *Aedes* species. ns: *p* > 0.05.

## Data Availability

The data (Online dataset 1) that support the findings of this study are available at https://doi.org/10.6084/m9.figshare.23584812.

## Supplementary Materials

The following supporting information can be downloaded at: https://www.mdpi.com/article/10.3390/insects14100810/s1, Table S1: Correlations among the 32 predictors; Table S2: Importance values of predictors in the preliminary species distribution models; Table S3: Comparisons of TSS and AUC between the real and null species distribution models; Table S4: Importance values of the predictors calibrated by eight algorithms; Table S5: AUC and TSS calibrated by eight algorithm.
