# Supplementary material for: Niche and Range Shifts of Aedes aegypti and Ae. albopictus Suggest That the Latecomer Shows a Greater Invasiveness
Source: Insects. 2023 Oct 13;14(10):810. doi: 10.3390/insects14100810 (PMC10607146; doi:10.3390/insects14100810)
Supplement: Supplementary file 1 [file insects-14-00810-s001.zip › S1.pdf]

S1 Importance value of each predictors in the preliminary ecological niche models

predictors	AAE (Introduced)	AAE (Native)	AAL (Introduced)	AAL (Native)
Annual mean temperature	0.107	0.149	0.116	0.167
Mean diurnal range(mean of monthly(max temp-min temp))	0.018	0.041	0.034	0.029
Isothermality(bio2/bio7)(×100)	0.074	0.394	0.113	0.071
Temperature seasonality (standard deviation ×100)	0.288	0.221	0.185	0.216
Max temperature of the warmest month	0.064	0.037	0.115	0.047
Min temperature of the coldest month	0.097	0.113	0.132	0.088
Temperature annual range(bio5-bio6)	0.106	0.095	0.105	0.056
Mean temperature of the wettest quarter	0.050	0.054	0.033	0.080
Mean temperature of the driest quarter	0.095	0.056	0.047	0.062
Mean temperature of the warmest quarter	0.147	0.143	0.270	0.048
Mean temperature of the coldest quarter	0.255	0.145	0.085	0.188
Annual precipitation	0.055	0.042	0.138	0.169
Precipitation of the wettest month	0.080	0.061	0.159	0.158
Precipitation of the driest month	0.028	0.022	0.041	0.034
Precipitation seasonality	0.021	0.018	0.014	0.026
Precipitation of the wettest quarter	0.046	0.052	0.102	0.112
Precipitation of the driest quarter	0.041	0.014	0.070	0.066
Precipitation of the warmest quarter	0.021	0.019	0.032	0.181
Precipitation of the coldest quarter	0.023	0.010	0.021	0.035
Gross domestic product	0.161	0.214	0.166	0.059
Density of population	0.115	0.128	0.112	0.159
Fractions of cropland	0.060	0.037	0.046	0.044
Fractions of managed pasture	0.006	0.012	0.040	0.057
Fractions of forested primary land	0.012	0.022	0.018	0.019
Fractions of non-forested primary land	0.013	0.026	0.019	0.008
Fractions of rangeland	0.025	0.041	0.031	0.091
Fractions of potentially forested secondary land	0.007	0.011	0.006	0.006
Fractions of potentially non-forested secondary land	0.009	0.017	0.006	0.010
Fractions of urban	0.011	0.005	0.017	0.003
Aspect	0.011	0.011	0.010	0.011
Elevation	0.016	0.030	0.028	0.030
Slope	0.011	0.017	0.015	0.016

Note: AAE: *Aedes aegypti*; AAL: *Aedes albopictus*.
