# Supplementary material for: Niche and Range Shifts of Aedes aegypti and Ae. albopictus Suggest That the Latecomer Shows a Greater Invasiveness
Source: Insects. 2023 Oct 13;14(10):810. doi: 10.3390/insects14100810 (PMC10607146; doi:10.3390/insects14100810)
Supplement: Supplementary file 1 [file insects-14-00810-s001.zip › S3.pdf]

S3 Comparisons of AUC and TSS between real and null ecological niche models.

Aedes aegypti (Introduced)			*Aedes aegypti* (Native)			*Aedes albopictus* (Introduced)			*Aedes albopictus* (Native)		
Models	AUC	TSS	Models	AUC	TSS	Models	AUC	TSS	Models	AUC	TSS
Real	0.984	0.876	Real	0.993	0.936	Real	0.978	0.852	Real	0.978	0.816
Real	0.983	0.878	Real	0.993	0.923	Real	0.979	0.854	Real	0.852	0.504
Real	0.985	0.881	Real	0.90	0.921	Real	0.978	0.852	Real	0.979	0.818
Real	0.984	0.881	Real	0.992	0.929	Real	0.979	0.852	Real	0.854	0.507
Real	0.984	0.880	Real	0.990	0.917	Real	0.979	0.858	Real	0.978	0.819
Null	0.822	0.503	Null	0.889	0.620	Null	0.816	0.504	Null	0.852	0.513
Null	0.821	0.489	Null	0.874	0.611	Null	0.818	0.507	Null	0.979	0.814
Null	0.819	0.497	Null	0.856	0.562	Null	0.819	0.513	Null	0.852	0.494
Null	0.813	0.459	Null	0.895	0.661	Null	0.814	0.494	Null	0.979	0.820
Null	0.820	0.490	Null	0.851	0.558	Null	0.820	0.513	Null	0.858	0.513

Paired sample *t* tests showed that all of $P < 0001$, and all of AUC and TSS in the null models were significantly lower than those in the real models.
